# From Media Literacy to Health Policy: Innovative Approaches to Safeguarding the Online Space from Sexual Health Misinformation: A Policy Brief

**DOI:** 10.34172/hpp.45710

**Published:** 2026-08-21

**Authors:** Sanam Borji-Navan, Leila Doshmangir, Mojgan Mirghafourvand

**Affiliations:** ^1^Student Research Committee, School of Nursing and Midwifery, Shahroud University of Medical Sciences, Shahroud, Iran; ^2^Department of Health Policy & Management, Tabriz Health Services Management Research Center, School of Management and Medical Informatics, Tabriz University of Medical Sciences, Tabriz, Iran; ^3^Social Determinants of Health Research Center, Tabriz University of Medical Sciences, Tabriz, Iran

**Keywords:** Information literacy, Health policy, Social media, Health communication, Sexual health, Health promotion

## Abstract

**Introduction:**

The digital space is the primary source of health information for peoples, yet it is saturated with sexual health misinformation. This infodemic poses a direct threat to public health. Current interventions are proving insufficient against this challenge, creating a need for innovative and comprehensive health policy.

**Methods:**

This policy brief was developed using the evidence-informed framework described by Lavis et al., following a four-step process: prioritization of the issue, problem identification, formulation of policy options, and identification of implementation considerations. High-level evidence was derived from an umbrella review evaluating digital intervention efficacy and from preliminary findings of a scoping review mapping educational gaps (currently under peer review).

**Results:**

The evidence synthesis reveals critical systemic deficits, primarily: an engagement gap, a content gap, and a trust deficit. Traditional interventions fail to compete with misinformation due to these structural limitations. To operationalize a secure digital ecosystem, this brief proposes five actionable policy options: (1) Establishing a Digital Health Innovation Fund; (2) Enforcing a Mandatory National e-Health Trust Certification; (3) Revising National Guidelines; (4) Institutionalizing a Specialized Governance Body; and (5) Implementing a National Digital Health Literacy Program.

**Conclusion:**

By operationalizing these evidence-based options, policymakers can transform the digital space from a source of risk into a reliable infrastructure for health promotion, serving as a replicable model for other nations facing similar challenges.

## Introduction

 Approximately 19% of new HIV diagnoses occur among individuals aged 13–24, and 53% of new sexually transmitted infections are reported in the 15–24 age group.^1,2^ Traditional barriers such as stigma and lack of youth-friendly services prevent adolescents from accessing care. Digital interventions offer a unique solution to bypass these barriers, providing anonymity and accessibility that traditional clinics cannot offer.^2^

 In an era where the majority of young adults seek health information online,^3^ the online environment has become a critical, yet hazardous, public square.^4^ Online environments are currently saturated with sexual health misinformation, characterized by inaccurate, misleading, and often harmful content that proliferates significantly faster than factual information.^4,5^ This infodemic constitutes a direct and escalating threat to the well-being of the current generation, compromising decades of advancements in public health.^6^

 The ramifications of failure to intervene are profound. Unmitigated health misinformation precipitates suboptimal decision-making, exacerbates high-risk behaviors, delays necessary medical care, and imposes a substantial economic burden on public health systems.^7,8^ Furthermore, it fosters an entrenched distrust in public health authorities, significantly impeding the uptake of future interventions ranging from vaccination campaigns to STI prevention efforts.^7-9^

 While individual media literacy remains a necessary foundation, it is insufficient in isolation. Effective public protection requires a paradigm shift from passive education to active, systemic health policy. This brief proposes innovative, evidence-based strategies to build a resilient regulatory framework that ensures platform accountability and protects the integrity of digital health information.

## Methods

 This policy brief was developed using the evidence-informed framework described by Lavis et al.^10^. The development process followed a systematic approach comprising four key phases: prioritization of the policy issue, clarification of the problem, framing of policy options, and identification of implementation considerations.^10^

 To ensure the recommendations are grounded in robust scientific evidence, we adopted a targeted evidence synthesis approach. Evidence was derived primarily from two recent reviews conducted by Borji et al.^2,11^. The scoping review cited in this brief is currently under peer review; therefore, its findings should be considered preliminary. Its methodology is supported by a published protocol.^12^ Together, these sources map the digital sexual health landscape.

 The first source was a scoping review,^11^ that utilized a search strategy with key concepts (Population, Intervention, and Outcome) across databases of Web of Science, PubMed, Scopus, EMBASE, and the Cochrane Library and search engines like Google Scholar, Semantic Scholar, and Elmnet to map the dimensions of digital education.^11^ The second source was an umbrella review (2024), which synthesized meta-level evidence from systematic reviews regarding intervention efficacy.^2^ This study utilized a search strategy with three broad categories (Sexual health and related issues, digital health interventions, and adolescent) across databases of the search engines and databases of PubMed, Web of Science, Scopus, Chochrane Library, Google Scholar.

 These pivotal studies were selected because they provide the most up-to-date and context-specific data available on the target demographic. The scoping review (preliminary findings) specifically addresses content characteristics,^11^ while the umbrella review offers high-certainty evidence on methodological effectiveness.^2^

 Evidence was extracted at the review level; the primary studies included in the scoping review were not re-analyzed. Key findings related to intervention characteristics, engagement strategies, content focus, and governance mechanisms were systematically reviewed and compared. Through an interpretive thematic synthesis of the reported findings across the two reviews, three recurrent structural gaps were identified: engagement gap, content gap, and trust deficit.

 The methodological quality of the systematic reviews included in the umbrella review had been previously assessed using the AMSTAR-2 tool. The appraisal indicated that most reviews were of moderate (n = 16) or high quality (n = 5), with only a small number rated as low quality (n = 2).^2^ Therefore, no additional quality appraisal was conducted for this policy brief; however, the methodological quality reported in the umbrella review was considered when interpreting the evidence.

 Initially, the mitigation of sexual health misinformation ^4,5^ was prioritized as a critical policy issue due to its escalating impact on public health outcomes.^6-8^

 To clarify the problem and its context, specific barriers were identified through a preliminary review of current digital health trends.^11,12^

 To frame policy options, we synthesized high-level evidence from two key reviews.^2,11^ These findings informed the formulation of policy solutions tailored to address the identified content gaps.

 Finally, keeping with the framework’s emphasis on applicability, the proposed options were assessed for implementation considerations rather than simple prioritization. We mapped the options to the local context to evaluate feasibility, potential barriers, and ethical implications.

## Results

 As illustrated in the conceptual framework(Figure 1), the evidence for this policy brief was synthesized from two reviews,^2,11^ representing a total of 74 primary studies.

**Figure 1:**
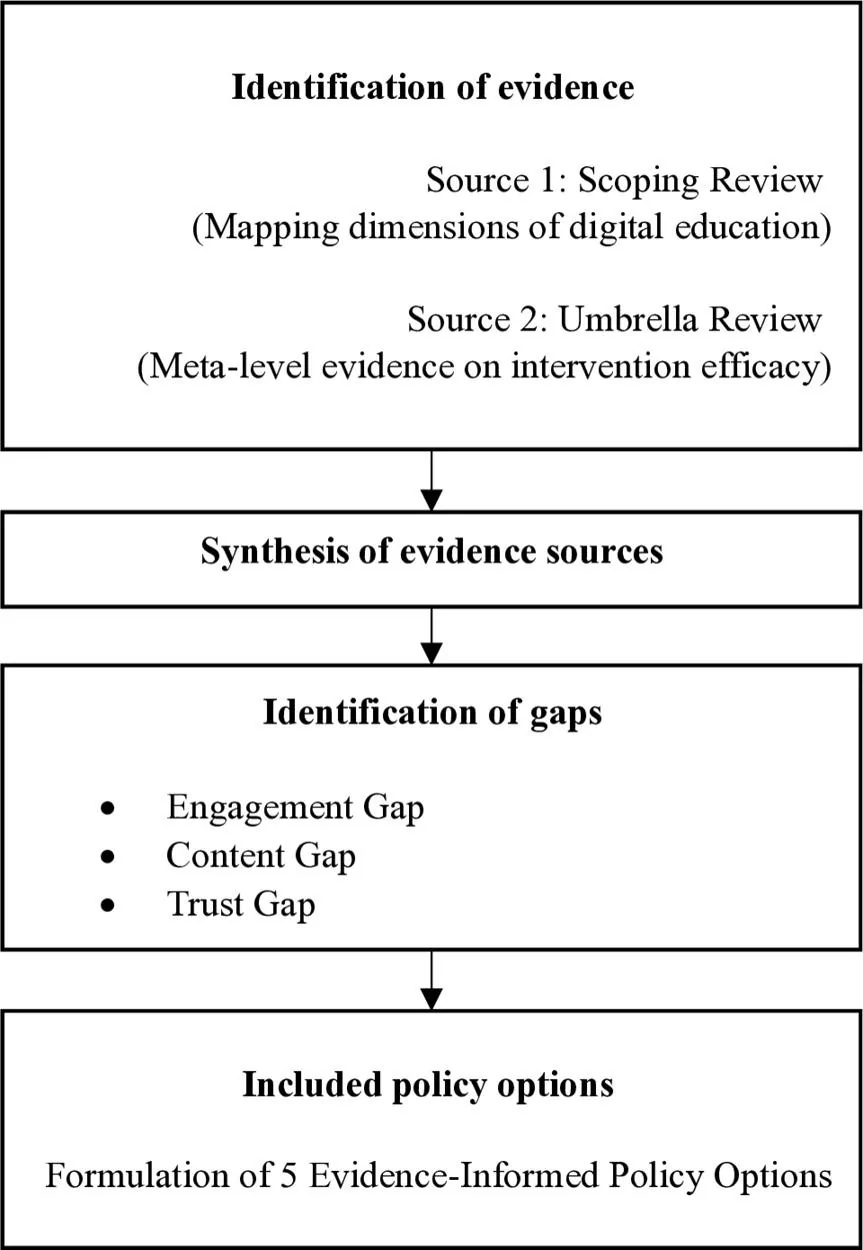
Conceptual process of evidence selection and policy option formulation

 The study selection processes for the scoping review and umbrella review are presented in Figures 2 and 3, respectively.

**Figure 2:**
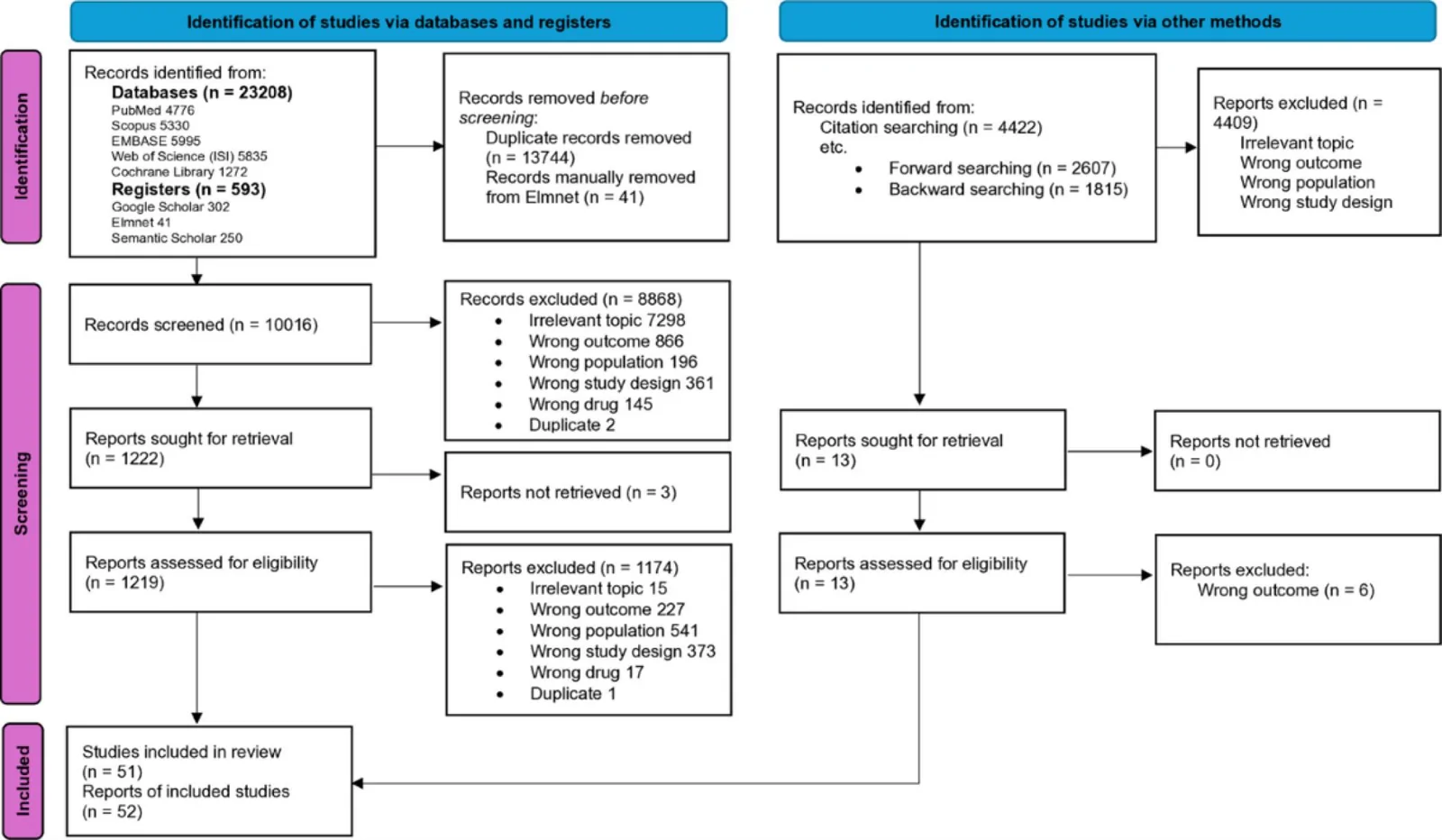
PRISMA flow diagram (Scoping review). Source: Borji et al.^11^

**Figure 3:**
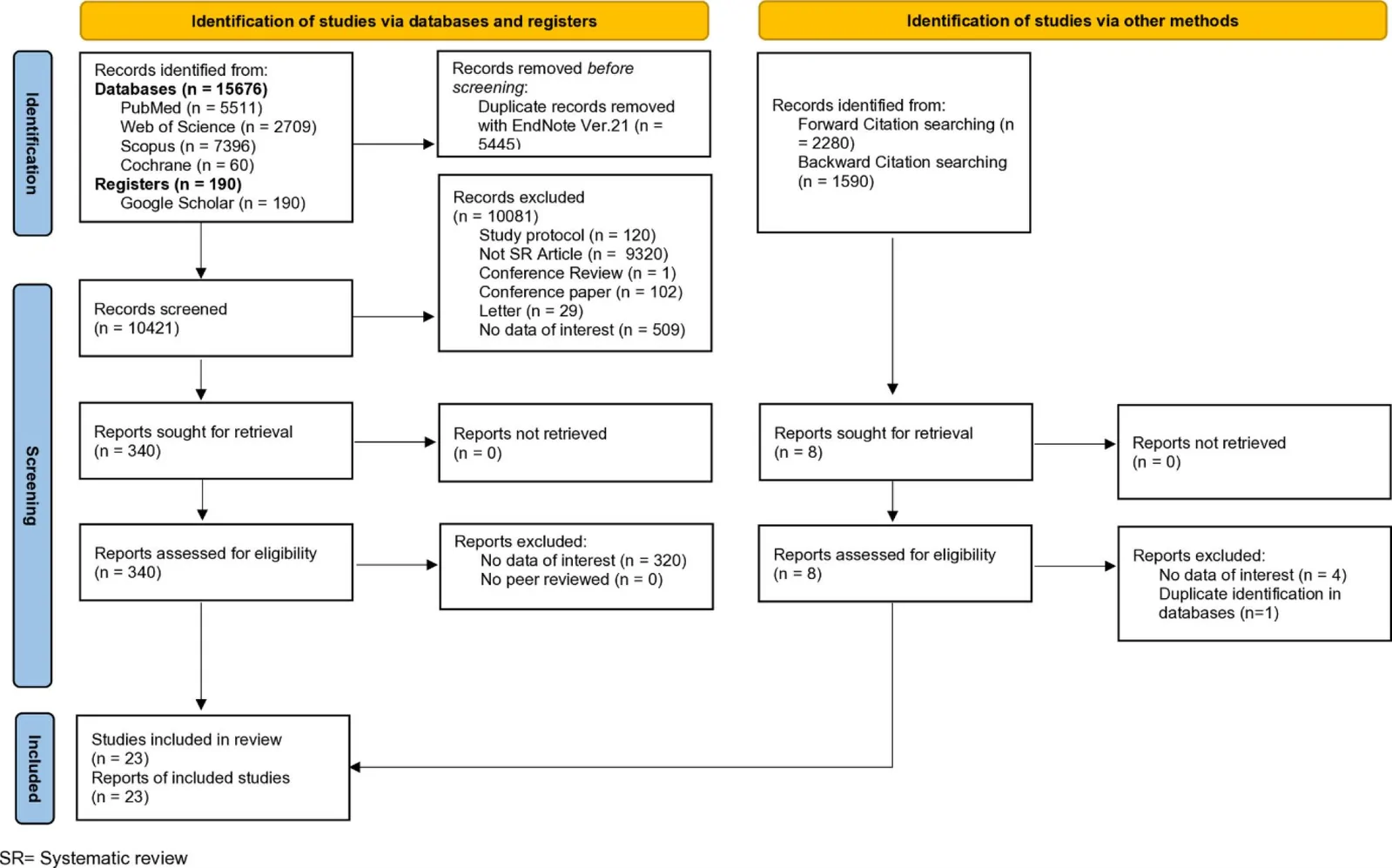
PRISMA flow diagram (Umbrella review). Source: Borji et al.^2^

 The analysis of these datasets allowed for a mapping of the current digital sexual health landscape, identifying structural deficiencies in content, delivery, and governance. In line with the status of the scoping review as a work currently under peer review, the findings derived from this source should be interpreted as preliminary and subject to potential changes upon final publication. The synthesis highlighted three critical gaps necessitating targeted policy interventions:

 First, a pronounced engagement gap is evident within the digital health landscape. While traditional text-based interventions, such as SMS,^13,14^ demonstrate efficacy for discrete clinical endpoints, they are often insufficient to sustain the longitudinal engagement necessary for complex behavioral modification. In contrast, interactive modalities, particularly gamified applications,^15-19^ have been shown to significantly enhance self-efficacy and user retention among the youth demographic.^11^

 Furthermore, a substantial discrepancy exists between user requirements and official content strategies. Existing institutional interventions predominantly prioritize a biomedical and pathology-oriented framework. The absence of holistic content addressing key topics such as healthy relationships and consent creates a critical gap, leading adolescents to seek information from unverified and frequently misleading commercial source.^11^

 Finally, trust remains a primary determinant of intervention uptake. The analysis reveals that users exhibit significant reluctance to engage with platforms characterized by opaque data governance. Consequently, the target demographic frequently prioritizes anonymity over accuracy, favoring unreliable commercial channels over official platforms due to privacy apprehensions.^11^

###  Policy Options

 To bridge the gap between media literacy and health policy, several structural interventions are required. Table 1 summarizes the key evidence from the source reviews that directly supports each proposed policy option.

**Table 1:** Summary of evidence supporting the proposed policy options

**#**	**Policy Option**	**Key Findings / Supporting Evidence**
1	Establishment of a digital health innovation fund	There is a significant lack of culturally adapted interventions and studies in low-resource/LMIC settings. Funding is required to bridge this gap, support local innovations, and design platforms capable of sustaining long-term behavioral change.^2,11^
2	Implementation of a mandatory national e-Health trust certification	While digital health interventions effectively improve knowledge and reduce risky behaviors, long-term behavior change remains inconsistent. A certification system is necessary to ensure that available apps and platforms meet high-quality, evidence-based standards to combat misinformation effectively.^2^
3	Revision of national sexual health education guidelines	A review of 51 studies revealed that digital interventions predominantly focus on a disease-prevention model, specifically HIV/STIs prevention (n = 33) and safer sex practices (n = 26). Conversely, broader and rights-based topics, such as sexual identity, gender, and rights, are severely underrepresented (n = 3). This highlights the urgent need to revise guidelines to ensure comprehensive, inclusive, and rights-oriented content.^11^
4	Institutionalization of a Specialized Digital Health Governance Body	The digital health landscape is highly fragmented, employing diverse delivery vehicles such as web-based platforms (27%), SMS systems (24%), mHealth apps, and emerging interactive formats like gamification. A dedicated governance body is essential to oversee, standardize, and evaluate this diverse ecosystem effectively. While digital interventions successfully drive short-term knowledge gains and health screening uptake, their impact on long-term behavioral change (e.g., sustained condom use, vaccination rates) is inconsistent. Furthermore, qualitative evidence highlights that user engagement depends on features like personalization, anonymity, and cultural appropriateness. A specialized governance body is essential to establish long-term evaluation frameworks and standardize these interventions for sustained impact.^11^
5	Implementation of a National Digital Health Literacy Program	Digital interventions are proven to significantly enhance sexual health knowledge, improve health literacy, and increase the uptake of screening services. A national program would scale these validated benefits to empower youth to safely navigate online spaces.^2,11^

 To effectively address the three critical deficiencies identified in the evidence synthesis, five distinct policy options were formulated. These options were derived from the findings of the included reviews.^2,11^ For every identified structural barrier, a corresponding policy intervention was designed to mitigate it. The proposed options were then prioritized based on their potential public-health impact, feasibility within the current national infrastructure, and their structural interdependencies. Based on interdependencies among the options, we propose a phased implementation sequence in which Option 4 (governance) precedes the development of standards (Option 2) and the certification mechanism (Option 5), forming a core foundational package. Options 1 and 3 can follow once the governance and standards infrastructure is established.

 Below is an analysis of the five proposed options, including their potential advantages and implementation challenges. These options represent actionable administrative and regulatory measures designed to transition the current landscape from a restrictive approach to a supportive digital ecosystem.

####  Option 1: Establishment of a Digital Health Innovation Fund

 Instead of general funding for static websites, the Ministry of Health should allocate specific grants dedicated to immersive technologies. This policy entails redirecting financial resources toward the development of AI-driven and gamified tools that can compete with misinformation in terms of user experience (UX) and interactivity. The primary advantage of this strategy is that it directly incentivizes the creation of high-retention platforms, aligning national interventions with global trends in youth-friendly digital health. However, the requirement for significant initial financial investment and the need for ongoing technical maintenance (which typically requires allocating 15% to 20% of the initial development costs annually),^20,21^ may strain limited public sector budgets.

####  Option 2: Implementation of a Mandatory National e-Health Trust Certification

 To standardize quality, regulatory bodies must enforce a certification system. This policy option requires apps to meet strict end-to-end encryption and scientific accuracy standards to receive a government-endorsed trust certification, creating a visible trusted environment for users. This approach offers the critical benefit of creating a visible safe zone, allowing users to ensuring data privacy. Conversely, a barrier to implementation is resistance from developers due to strict compliance costs, alongside the need for a robust technical infrastructure for continuous monitoring and auditing.

####  Option 3: Revision of National Sexual Health Education Guidelines 

 This option involves a regulatory update to the national health curriculum and online content standards. It mandates the inclusion of holistic and psychosocial topics in all state-approved digital interventions to ensure content meets the actual needs of the target demographic. The strategic merit of this option lies in ensuring content relevance, thereby filling the information void that currently drives adolescents toward unverified commercial sources. Nevertheless, necessitating extensive training for content creators and educators to align with the new standards.

####  Option 4: Institutionalization of a Specialized Digital Health Governance Body

 To ensure sustainability and accountability, a dedicated national task force or steering committee should be established. This centralized entity would act as the primary steward for digital sexual health, responsible for orchestrating inter-sectoral collaboration, overseeing the certification process, and monitoring the long-term impact of digital interventions on public health outcomes. The distinct advantage of this governance model is that it ensures centralized accountability and prevents the fragmentation of efforts across different organizations. However, the establishment of such a body faces the challenge of requires a clear mandate to effectively coordinate between diverse stakeholders, such as the Ministries of Health, Education, and Information and Communications Technology (ICT).

####  Option 5: Implementation of a National Digital Health Literacy Program

 To address the demand side of the information ecosystem, this policy mandates the integration of digital health literacy into public education and community outreach programs. The objective is to equip the target demographic with the critical skills necessary to evaluate the credibility of online sexual health sources, thereby reducing susceptibility to algorithmic misinformation. This policy empowers users to independently navigate the digital space, providing a sustainable defense against misinformation regardless of platform changes. Yet, it must be noted that this is a long-term strategy; results will be gradual rather than immediate, and curriculum integration can be a slow process.

Table 2 presents a prioritization matrix evaluating the five proposed policy options based on their potential impact, technical and political feasibility, financial cost, and estimated time to implementation.

**Table 2:** Prioritization Matrix for Proposed Policy Options

**#**	**Policy Option**	**Impact**	**Feasibility**	**Cost**	**Time to Implementation**
1	Establishment of a digital health innovation fund	High	Medium	High	Long-term
2	Implementation of a mandatory national e-Health trust certification	High	Medium	Medium	Medium-term
3	Revision of national sexual health education guidelines	Medium	High	Low	Short-term
4	Institutionalization of a Specialized Digital Health Governance Body	High	Low	High	Long-term
5	Implementation of a National Digital Health Literacy Program	Medium	High	Medium	Short-term

###  Implementation Considerations

 Implementation of the proposed options depends on feasibility, anticipated barriers, and their structural interdependencies. A phased approach is therefore required. Establishing a central governance body (Option 4) is the most immediately feasible step, leveraging existing institutional structures. Developing national standards (Option 2) and a certification mechanism (Option 5) requires technical capacity and sustained cross-sectoral coordination. Barriers include potential resistance from developers, limited public-sector resources, and disparities in digital access that may constrain the effectiveness of literacy and curriculum-related interventions (Options 1 and 3). All options must align with national data-protection principles, with special attention to avoiding widened inequities for adolescents with limited digital access.

 Furthermore, ethical considerations and safeguards must be centrally integrated into the rollout. While the mandatory certification system (Option 2) aims to protect users, it risks introducing unintended harms such as government overreach, privacy violations, and the exclusion of valuable, grassroots community sources that lack the financial or technical resources to undergo formal certification. To mitigate these risks, the governance body must ensure that the certification criteria are fully transparent, strictly limited to verifying scientific accuracy and data security (without monitoring individual user activity), and include accessible, tiered evaluation pathways to support non-profit and community-driven platforms.

 Furthermore, successful execution requires a clear understanding of the involved actors and potential friction points. Table 3 outlines a stakeholder analysis, identifying the lead organizations for each policy option and the anticipated sources of resistance.

**Table 3:** Stakeholder Analysis and Potential Resistance

**#**	**Policy Option**	**Lead Actors**	**Potential Points of Resistance**
1	Establishment of a digital health innovation fund	Ministry of Health; Ministry of Technology/Economy; Private Investors	Fiscal conservatives (budget constraints); tech startups (bureaucratic funding processes).
2	Implementation of a mandatory national e-Health trust certification	Ministry of Health; National Medical Councils	Platform owners (compliance costs); public/digital rights groups (concerns over censorship or privacy).
3	Revision of national sexual health education guidelines	Ministry of Health; Academic and Research Institutions	Conservative cultural or political groups (due to sensitivities surrounding sexual health topics).
4	Institutionalization of a Specialized Digital Health Governance Body	Ministry of ICT^*^; Ministry of Health; Judiciary	Existing regulatory agencies (turf wars/loss of jurisdiction); civil rights organizations (over-regulation fears).
5	Implementation of a National Digital Health Literacy Program	Ministry of Education; Public Health NGOs^†^; Mass Media	Educational boards (curriculum overload); teachers (lack of specialized training).

ICT: Information and Communications Technology; ^†^NGOs: Non-governmental organization.

 To ensure coherence and reduce implementation risks, a staged rollout is recommended:

1. Option 4 – Governance body: foundational coordination and oversight.
2. Option 2 – National standards: developed under the governance structure.
3. Option 5 – Certification mechanism: operationalized once standards exist.
4. Options 1 and 3 – Innovation funding and curriculum reform: introduced after the regulatory framework is established.

## Discussion

 This policy brief advocates for a fundamental paradigm shift in managing digital sexual health: moving from a framework of individual responsibility to one of systemic accountability. Our findings underscore that the failure of current interventions stems not from a scarcity of medical information, but from structural deficits in engagement mechanisms, content relevance, and institutional trust.

 To address these deficits, the proposed digital ecosystem approach was evaluated against two prevailing models identified in global strategies: the restrictive model and the literacy-only model.^22-25^ While many nations rely on filtering or blocking misinformation,^26^ our analysis suggests this method is reactive and ultimately insufficient against the high velocity of myths.^27-29^ Furthermore, restrictive measures often yield counterproductive results, inadvertently driving users toward channels where misinformation proliferates unchecked.^30^ Similarly, while digital literacy is vital, relying solely on users to distinguish credible information places an undue cognitive burden on individuals who remain vulnerable to sophisticated algorithmic manipulation.^31,32^ In contrast, the recommended ecosystem approach addresses the root cause by saturating the digital space with high-quality, scientifically accurate, and engaging alternatives.

 The justification for the proposed policy options is rooted in the synthesized evidence. The recommendation to establish a digital health innovation fund (Option 1) directly addresses the engagement gap highlighted, acknowledging that static content lacks the retention power of gamified interventions.^2,11^ Concurrently, the proposal for a mandatory e-Health trust certification (Option 2) targets the trust deficit. Unlike voluntary guidelines, a state-endorsed certification creates a visible differentiator, allowing evidence-based platforms to compete effectively with anonymous commercial sources.^2,11^ Crucially, the inclusion of a governance body (Option 4) as the initial step, followed by national standards (Option 2) and a certification mechanism (Option 5), forms the foundational package upon which the other measures rest. The inclusion of holistic guidelines (Option 3) ensures that these initiatives are not transient, but are sustained through centralized stewardship and responsiveness to user needs.^2,11^

 The adoption of these recommendations carries profound multi-level implications. From a public health perspective, the transition from passive information dissemination to active skills-building (via Option 5) is expected to mitigate high-risk behaviors. Economically, this represents a strategic pivot from costly treatment models to cost-effective digital prevention. Most critically, establishing secure, state-sanctioned platforms will help restore the eroded trust between the youth population and public health authorities, ensuring that valid science reclaims its position as the dominant voice in the digital sphere.

## Conclusion

 The unmitigated proliferation of sexual health misinformation constitutes a critical determinant of public health, exacerbating risky behaviors and widening the disconnect between adolescents and credible care systems. This policy brief demonstrates that the prevailing reliance on static, pathology-oriented, and unregulated platforms is insufficient to address the evolving needs of the digital-native demographic.

 Drawing upon a synthesis of high-level evidence, this study proposes a comprehensive digital ecosystem strategy founded on five strategic imperatives. To reform the supply side, the policy prioritizes the establishment of a digital health innovation fund and the enforcement of mandatory trust certification protocols alongside updated content guidelines. Crucially, to ensure sustainability and user empowerment, the framework mandates the institutionalization of a specialized governance body for centralized stewardship and the implementation of a national digital health literacy program.

 The systemic operationalization of these evidence-informed policy options offers a viable pathway to reconfigure the digital environment from a domain of risk into a robust infrastructure for health promotion. Ultimately, the successful deployment of this secure, and engaging ecosystem could serve as a replicable model for nations navigating the complexities of the global infodemic, demonstrating how evidence-based governance can effectively safeguard public health.

## Competing Interests

 The authors declare that they have no competing interests.

## Ethical Approval

 Not applicable.
